# Bioinformatics-based identification of differentially expressed genes in endometrial carcinoma: implications for early diagnosis and prognostic stratification

**DOI:** 10.3389/fgene.2025.1631060

**Published:** 2025-09-17

**Authors:** Liang Gao, Donglan Yuan, Aihua Huang, Hua Qian

**Affiliations:** Department of Gynecology, The Affiliated Taizhou People’s Hospital of Nanjing Medical University, Taizhou School of Clinical Medicine, Nanjing Medical University, Taizhou, China

**Keywords:** bioinformatics analysis, diagnosis, prognosis, endometrial carcinoma, biomarkers

## Abstract

**Introduction:**

This study aims to identify differentially expressed genes (DEGs) in endometrial carcinoma (EC) through bioinformatics analysis and investigate their roles in early diagnosis and prognosis.

**Methods:**

EC-related gene datasets were retrieved from the NCBI and analyzed using R packages to screen for DEGs. Primers were designed for selected DEGs, and their expression levels were validated via qPCR. Logistic regression, survival analysis, Cox proportional hazards models, and random forest models were employed to evaluate associations between DEGs and clinical outcomes.

**Results:**

Bioinformatics analysis identified significantly upregulated genes (*Erb-B2*, *PIK3CA*, *CCND1*, *VEGF*, *KIT*) and downregulated genes (*PTEN*, *E-cadherin*, *p53*). Logistic regression revealed *Erb-B2* as a protective factor against poor prognosis, whereas *E-cadherin* and *P53* were risk genes. Clinical markers CA125, CA199, and IL-9 also emerged as prognostic risk factors. Survival analysis demonstrated significant divergence between good and poor prognosis groups (*P* < 0.05), with HR < 1 for *Erb-B2* and *p53* (protective effects) and HR > 1 for *E-cadherin*, CA125, CA199, and IL-9 (risk effects). The random forest model highlighted CA199 as a pivotal prognostic biomarker, while decision tree analysis enabled effective patient stratification based on CA125 and CA199 thresholds.

**Conclusion:**

The identified DEGs and clinical indicators hold significant potential for improving early diagnosis and prognostic evaluation in EC. These findings provide novel biomarkers and theoretical foundations for precision medicine, guiding risk stratification and personalized therapeutic strategies.

## 1 Introduction

Endometrial carcinoma (EC), one of the three most prevalent malignancies in the female reproductive system, has demonstrated a rising global incidence, posing a significant threat to women’s health and quality of life (Cai et al., 2021). Early diagnosis and accurate prognostic evaluation are critical for improving survival outcomes in EC patients, as they enable clinicians to tailor individualized therapeutic strategies while avoiding overtreatment or undertreatment. However, current diagnostic modalities for early-stage EC are limited by invasiveness, low patient acceptance, and the frequent absence of overt symptoms in early phases, leading to underdiagnosis. Although existing prognostic markers hold clinical utility, their predictive capacity for individual outcomes remains insufficient to meet the demands of precision medicine (Zheng et al., 2025). The rapid advancement of genomic technologies has unlocked substantial potential for bioinformatics in cancer research (Zeng et al., 2021). By systematically mining and analyzing large-scale gene expression datasets, bioinformatics approaches can identify differentially expressed genes (DEGs) associated with tumorigenesis, progression, and prognosis. These DEGs may serve as potential biomarkers, offering novel avenues for early detection and prognostic stratification in EC (Yang et al., 2025). This study aims to leverage bioinformatics tools to screen DEGs with significant expression alterations in EC and investigate their roles in early diagnosis and prognostic evaluation. The findings are expected to provide scientific evidence and theoretical support for advancing precision diagnostics and therapeutics in EC.

## 2 Materials and methods

### 2.1 General characteristics

A total of 202 patients diagnosed with EC between January 2017 and January 2020 were retrospectively enrolled and stratified into a good prognosis group (n = 129) and a poor prognosis group (n = 73) based on clinical outcomes. No significant differences in baseline demographic or clinical characteristics were observed between the groups (*P* > 0.05; Table 1). However, there was a difference in the distribution of each type between the good prognosis group and the poor prognosis group (P = 0.013; Table 1), and the CNH type accounted for a higher proportion in the poor prognosis group (57.53% vs. 35.66%) This study was approved by the Ethics Committee of The Affiliated Taizhou People’s Hospital of Nanjing Medical University, and written informed consent was obtained from all participants or their families.

**TABLE 1:** General information.

		Good prognosis (n = 129)	Poor prognosis (n = 73)	*X* ^*2*^	*P*
Hypertension	Yes	59 (45.74)	30 (41.1)	0.407	0.523
	No	70 (54.26)	43 (58.9)		
Diabetes	Yes	63 (48.84)	43 (58.9)	1.894	0.169
	No	66 (51.16)	30 (41.1)		
Reproductive history	Yes	73 (56.59)	33 (45.21)	2.422	0.12
	No	56 (43.41)	40 (54.79)		
Family history	Yes	21 (16.28)	15 (20.55)	0.58	0.446
	No	108 (83.72)	58 (79.45)		
Smoking	Yes	13 (10.08)	8 (10.96)	0.039	0.844
	No	116 (89.92)	65 (89.04)		
Alcohol Consumption	Yes	39 (30.23)	21 (28.77)	0.048	0.827
	No	90 (69.77)	52 (71.23)		
	POLE mutation	15	3	10.737	0.013
Molecular typing	microsatellite instability	30	15		
	copy number low	38	13		
	copy number high	46	42		
Age (year)		46.02 ± 13.28	48.59 ± 12.42	−1.363	0.174

Inclusion criteria comprised: (1) diagnosis of EC confirmed by postoperative pathological examination in accordance with the guidelines from the Diagnosis and Management of Endometrial Cancer (Braun et al., 2016); (2) availability of comprehensive clinical records; (3) stringent quality control (QC) of analytical data; (4) complete gene expression profiles covering all targets of interest without significant missing values or outliers; and (5) signed informed consent forms. Exclusion criteria included: (1) concurrent malignancies; (2) prior neoadjuvant therapies (e.g., chemotherapy, radiotherapy) that might alter tumor gene expression profiles; (3) suboptimal sample quality; and (4) incomplete follow-up data.

### 2.2 Bioinformatics analysis

The Gene Expression Omnibus (GEO) dataset GSE120490, comprising EC patient samples, was downloaded from the National Center for Biotechnology Information (NCBI) using the GEOquery package in R. Raw data were preprocessed and normalized, followed by gene identifier (ID) matching and differential expression analysis using the stringr, limma, and tidyverse packages. Gene Ontology (GO) enrichment analysis was performed with the clusterProfiler, org. Hs.e.g.,.db and enrichplot packages. Unsupervised dimensionality reduction using PCA was performed to verify the intrinsic expression differences between tumor tissue and normal tissue, while excluding technical batch effects. To further quantify the association between gene expression and sample grouping, a supervised dimensionality reduction model was constructed using partial least squares discriminant analysis (PLS-DA) (Mallardo et al., 2024). Using ‘tumor vs. normal’ or ‘good prognosis vs. poor prognosis’ as the dependent variable and DEGs expression level as the independent variable; Calculate R^2^ Y (model interpretability) and Q^2^ (predictive ability) through 10 fold cross validation to verify the reliability of the model. To refine biomarker candidates, feature selection was conducted via Least Absolute Shrinkage and Selection Operator (LASSO) regression, minimizing overfitting while identifying genes with the strongest prognostic relevance.

### 2.3 Indicator detection and follow-up

Lavage fluid in the uterine cavity was collected from patients and labeled for subsequent processing. Total cellular RNA was extracted using an RNA extraction kit (Tiangen Biotech Co., Ltd., China) and reverse-transcribed into complementary DNA (cDNA) with a reverse transcription (RT) kit. Primers were designed for target genes identified through prior screening, and quantitative polymerase chain reaction (qPCR) was performed using the synthesized cDNA as the template. Glyceraldehyde-3-phosphate dehydrogenase (GAPDH) served as the endogenous reference gene, while peripheral blood mononuclear cells (PBMCs) from healthy individuals undergoing routine physical examinations in our hospital were used as controls. Relative gene expression levels ≥ 2-fold compared to controls were defined as significant upregulation. Primer sequences are listed in Table 2. Genes exhibiting significant upregulation or downregulation (≥2-fold change) relative to healthy controls were assigned a binary value of 1, while others were coded as 0. Postoperative follow-up was conducted for 5 years, with patients experiencing recurrence or mortality classified into the poor prognosis group, and the remaining cases categorized into the good prognosis group.

**TABLE 2:** Primer sequences.

*Erb-B2*	Forward	5′GTG​AGG​CGG​GGT​GAA​GTC​CT 3′
	Reverse	5′GGC​ATC​GCT​CCG​CTA​GGT​GT 3′
*PIK3CA*	Forward	5′GAC​AAT​GAA​TTA​AGG​GAA​AA 3′
	Reverse	5′TGT​AGA​AAT​TGC​TTT​GAG​CT 3′
*CCND1*	Forward	5′TCA​TTT​CCA​ATC​CGC​CCT​CC 3′
	Reverse	5′CCT​CCT​CCT​CTT​CCT​CCT​CCT​C 3′
*VEGF*	Forward	5′CAT​CTT​CAA​GCC​GTC​CTG​TG 3′
	Reverse	5′CTC​GCT​CTA​TCT​TTC​TTT​GGT​C 3′
*KIT*	Forward	5′GCT​AGA​GCC​GGA​ACG​TGG​AAC​A 3′
	Reverse	5′AGG​AGC​AGC​AGA​ACG​AAG​AGG​AAA 3′
*PTEN*	Forward	5′AAA​GAC​ACT​ACG​ATG​CTG​CCA​AAT 3′
	Reverse	5′GCC​CTT​CCC​AGC​CTT​ACA​AT 3′
*E-cadherin*	Forward	5′CCA​GCT​TGG​GTG​AAA​GAG​TGA 3′
	Reverse	5′TTG​CTA​GGG​TCT​AGG​TGG​GTT​AT 3′

Peripheral venous blood samples (6 mL) were routinely collected from fasting patients preoperatively and centrifuged to isolate serum for subsequent analysis. Complete blood count parameters, including hemoglobin (Hb), white blood cell count (WBC), red blood cell count (RBC), and platelet count (PLT), were measured using an automated hematology analyzer. Serum levels of carbohydrate antigen 125 (CA125), carbohydrate antigen 199 (CA199), and interleukin-9 (IL-9) were quantified via chemiluminescence immunoassay (CLIA) following standardized protocols.

### 2.4 Statistical methods

Data processing and analyses were performed using SPSS Statistics 27.0 and R software version 4.4.3. Normally distributed continuous variables were expressed as mean ± standard deviation ( $\bar{x}$ ± s), while categorical variables were presented as n (%). Intergroup comparisons were conducted using independent samples t-tests (for continuous variables) and chi-square tests (for categorical variables). Multivariate binary logistic regression analysis was employed to identify independent risk factors, using variance inflation factor for collinearity screening. For gene feature selection, LASSO regression was implemented via the glmnet package in R. Kaplan-Meier (K-M) survival curves and Cox proportional hazards regression forest plots were generated using the forestplot, survminer, and survival packages in R. Random forest models were constructed with the randomForestSRC, ggRandomForests, pdp, and GGally packages to evaluate variable importance and partial dependence. Statistical significance was defined as a *P* < 0.05.

## 3 Results

### 3.1 DEG screening

**FIGURE 1:**
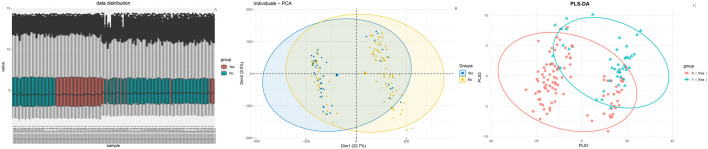
Data distribution **(A)** Box plot of gene expression distribution; **(B)** PCA plot of sample clustering (Group represents sample grouping in GSE120490: Yes = endometrial cancer tissue sample, No = normal endometrial tissue sample).

**FIGURE 2:**
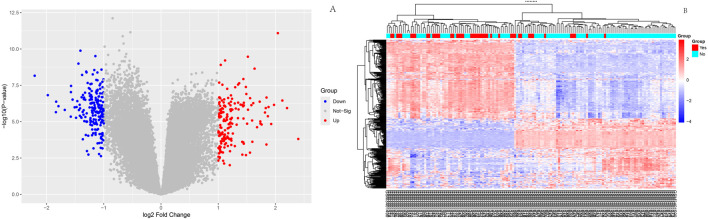
Differential expression analysis Volcano diagram: Red dots represent significantly upregulated genes, blue dots represent significantly downregulated genes, and gray dots represent non DEGs; This figure only reflects the differential expression of genes between tumors and normal tissues, and is not directly related to clinical prognosis outcomes; **(B)**:Heatmap of differentially expressed genes.

**FIGURE 3:**
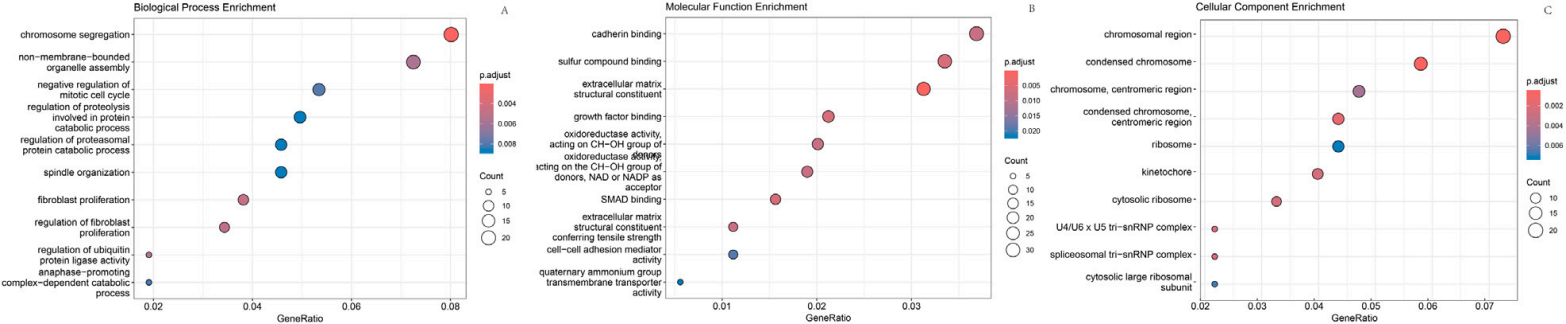
GO enrichment analysis. **(A)** Enriched biological processes; **(B)** Enriched molecular functions; **(C)** Enriched cellular components.

As shown in Figure 1, box plots (Figure 1A) and principal component analysis (PCA) plots of the GEO dataset GSE120490 (n = 145 samples) revealed no significant batch effects or inter-sample variability (*P* > 0.05). PCA analysis showed that the GSE120490 samples were clustered into two main clusters (Figure 1B), and the dataset included tumor tissue and normal tissue data. Further analysis confirmed that this grouping was driven by sample type (PCA1 contribution rate of 32.7%), rather than technical batch effects. Meanwhile, after correcting for batch effects using the SVA package in R language, the grouping trend remained significant, indicating biological differences. This is consistent with the inherent differences in gene expression profiles between tumor tissue and normal tissue. The PLS-DA (Figure 1C) score plot shows complete separation of tumor and normal tissue samples on the first principal component (explanatory power 41.2%) (R^2^ Y = 0.89, Q^2^ = 0.82), indicating a strong explanatory power of gene expression for sample types. Volcano plot (Figure 2A) was used to screen DEGs based on the statistical difference of gene expression level only, which was used to distinguish the abnormal expression genes in tumor tissues from normal tissues, and did not involve the association analysis with clinical prognosis outcome; The core genes related to prognosis were gradually screened through lasso regression (Section 2.2) and multiple regression (Section 2.5). Differential expression analysis (adjusted *P* < 0.05) identified 178 significantly upregulated genes, 200 downregulated genes, and 23,142 non-DEGs (Figure 2B). GO enrichment analysis demonstrated distinct functional annotations across biological processes (BP), molecular functions (MF), and cellular components (CC) (Figure 3).

### 3.2 LASSO regression for feature gene screening

**FIGURE 4:**
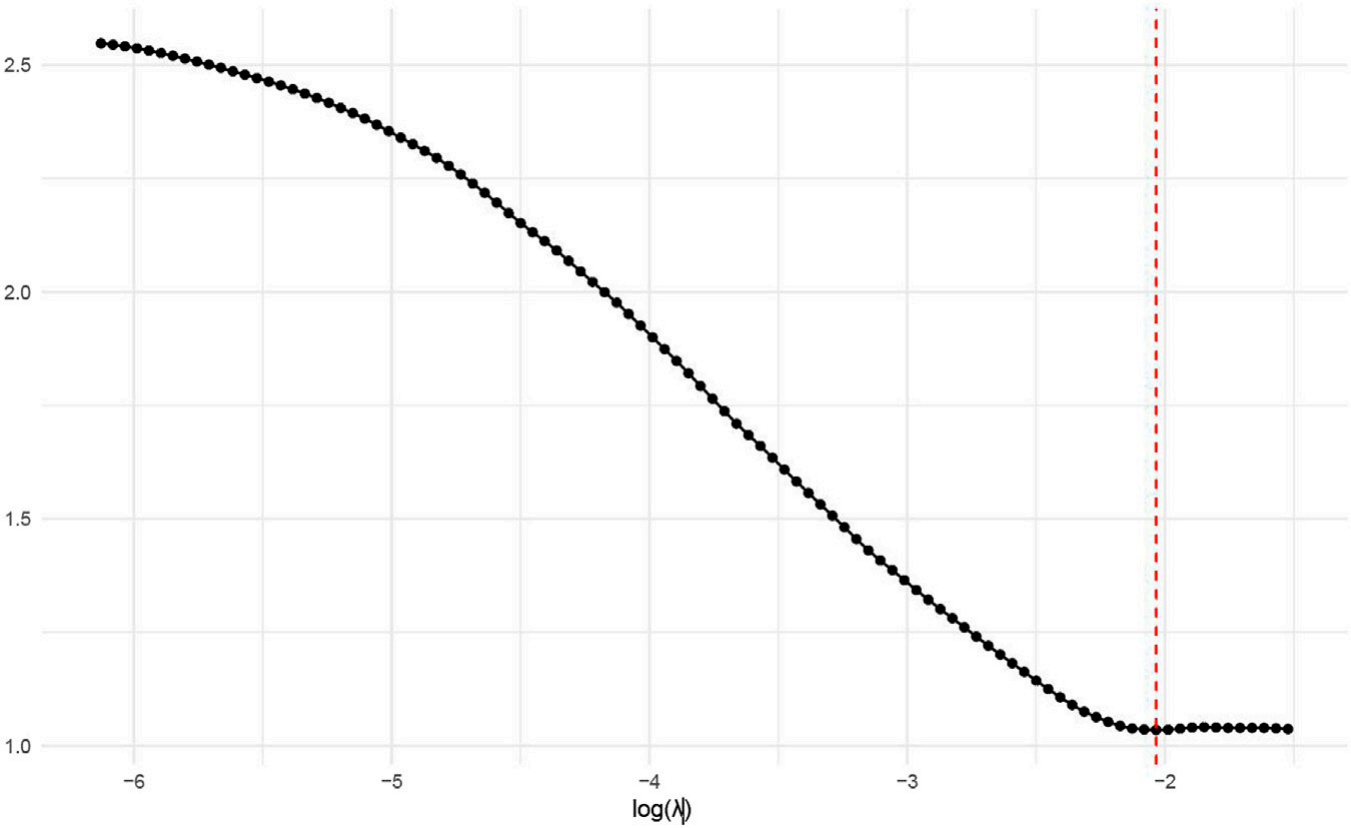
Relationship between log(λ) values and model error.

The 378 significant DEGs were subjected to LASSO regression to identify key predictive features. During variable selection, the penalty coefficient (λ) was systematically compressed across all 378 initial predictors. Optimal λ (λ = 0.013) was determined via cross-validation, minimizing the mean squared error while balancing model parsimony and goodness-of-fit. This process yielded a refined predictive model incorporating eight genes: *Erb-B2*, *CCND1*, *PIK3CA*, *VEGF*, *KIT*, *PTEN*, *E-cadherin*, and *p53* (Figure 4).

### 3.3 Comparison of gene expression and clinical indicators between groups

Based on qPCR results, gene expression changes were assessed by comparing each group’s data with healthy controls. Independent samples t-tests revealed significant differences in the expression of *Erb-B2*, *PIK3CA*, *VEGF*, *KIT*, *PTEN*, *E-cadherin*, and *p53* (*P* < 0.05), whereas *CCND1* showed no significant variation (*P* > 0.05). Similarly, tumor markers CA125 and CA199, RBC, and the inflammatory cytokine IL-9 exhibited statistically significant intergroup differences (Table 3).

**TABLE 3:** Differential gene expression and clinical indicator analysis.

Gene	Significance	Good prognosis (n = 129)	Poor prognosis (n = 73)	*X* ^*2*^	*P*
*Erb-B2*	Yes	12 (9.30)	57 (78.08)	98.059	<0.001
	No	117 (90.7)	16 (21.92)		
*PIK3CA*	Yes	47 (36.43)	43 (58.90)	9.528	0.002
	No	82 (63.57)	30 (41.10)		
*CCND1*	Yes	55 (42.64)	38 (52.05)	1.665	0.197
	No	74 (57.36)	35 (47.95)		
*VEGF*	Yes	34 (26.36)	31 (42.47)	5.543	0.019
	No	95 (73.64)	42 (57.53)		
*KIT*	Yes	39 (30.23)	45 (61.64)	18.935	<0.001
	No	90 (69.77)	28 (38.36)		
*PTEN*	Yes	107 (82.95)	14 (19.18)	78.921	<0.001
	No	22 (17.05)	59 (80.82)		
*E-cadherin*	Yes	97 (75.19)	16 (21.92)	53.686	<0.001
	No	32 (24.81)	57 (78.08)		
*p53*	Yes	84 (65.12)	11 (15.07)	46.873	<0.001
	No	45 (34.88)	62 (84.93)		
CA125(U/mL)		50.14 ± 3.48	52.25 ± 2.71	−4.484	<0.001
CA199(U/mL)		55.08 ± 3.03	58.36 ± 3.3	−7.144	<0.001
Hb(g/L)		132.07 ± 10.08	133.27 ± 9.96	−0.812	0.418
RBC(×10^12^/L)		4.91 ± 0.06	4.23 ± 0.33	23.067	<0.001
PLT (×10^9^/L)		205.13 ± 8.5	207.28 ± 8.19	−1.751	0.081
WBC(×10^9^/L)		4.58 ± 0.39	4.65 ± 0.3	−1.266	0.207
IL-9 (ng/L)		89.3 ± 8.73	103.2 ± 6.32	−11.946	<0.001

### 3.4 Subgroup analysis results

To explore the associations between molecular subtypes, clinical indicators, gene expression, and prognosis, subgroup analyses were performed based on four molecular subtypes of endometrial carcinoma (EC): POLE mutation (POLE), microsatellite instability (MSI), copy number low (CNL), and copy number high (CNH).No statistically significant differences were observed in clinical indicators [TIME, CA125, CA199, hemoglobin (Hb), red blood cell count (RBC), platelet count (PLT), white blood cell count (WBC), and interleukin-9 (IL-9)] among the four molecular subtypes (all P > 0.05; Table 4). Differential expression patterns of key genes (identified via LASSO regression) were observed across molecular subtypes, with two genes showing statistically significant associations. The proportion of ERBB2-positive cases varied significantly among subtypes (P = 0.0151),the CNL subtype had the lowest ERBB2 positivity (19%), while the POLE subtype showed the highest (50%); VEGF positivity also differed significantly across subtypes (P = 0.0103). The CNL subtype had the lowest VEGF positivity (17.5%), whereas the MSI subtype showed the highest (46.2%). For other genes (PIK3CA, CCND1, KIT, PTEN, E-cadherin, p53), no statistically significant differences in expression distribution were observed across subtypes (all P > 0.05), though trends were noted (e.g., CCND1 positivity was highest in MSI (56.4%) and lowest in POLE (30%); Table 4). Prognostic outcomes (good vs. poor) showed a trend across subtypes, though not statistically significant (P = 0.2721). The CNL subtype had the highest proportion of good prognosis cases (73%), while the CNH subtype had the lowest (57.8%). This is consistent with prior observations that CNH is overrepresented in the poor prognosis group (57.53% vs. 35.66% in good prognosis group).

**TABLE 4:** Results of multivariate binary logistic regression analysis.

Gene	B	Standard error	*W*	*P*	OR	95% CI	
						Lower limit	Upper limit
*Erb-B2*	−3.57	0.889	16.118	<0.001	0.028	0.005	0.161
*PIK3CA*	−0.353	0.768	0.212	0.646	0.703	0.156	3.162
*VEGF*	−0.361	0.818	0.195	0.659	0.697	0.14	3.465
*KIT*	−0.318	0.804	0.157	0.692	0.728	0.151	3.516
*E-cadherin*	2.207	0.841	6.881	0.009	9.088	1.747	47.277
*P53*	2.515	0.955	6.936	0.008	12.372	1.903	80.434
CA125	0.364	0.139	6.825	0.009	1.439	1.095	1.89
CA199	0.371	0.136	7.485	0.006	1.449	1.111	1.891
IL-9	0.258	0.08	10.316	0.001	1.294	1.106	1.515
Constant	−65.329	17.709	13.609	<0.001	0		

### 3.5 Multivariate binary logistic regression analysis

Factors demonstrating significant differences underwent collinearity diagnostics, revealing a variance inflation factor (VIF) > 5 for the gene *PTEN* and the clinical indicator RBC, indicating substantial multicollinearity. These variables were subsequently excluded from further analysis. Multivariate binary logistic regression analysis of the remaining factors identified *Erb-B2* as a protective factor against poor prognosis in EC patients, while *E-cadherin3* and *p53* emerged as risk factors for adverse outcomes. Clinical markers CA125, CA199, and IL-9 were also significantly associated with increased risk of poor prognosis (Table 5).

**TABLE 5:** Cox proportional hazards regression results.

Project	Regression coefficient	HR	Lower confidence interval	Upper confidence interval
CA125	0.02	1.03	0.96	1.1
CA199	0.06	1.06	0.99	1.13
IL-9	0.02	1.02	1	1.05
*Er-bB2*	−0.01	0.99	0.59	1.66
*E-cadhein*	0.04	1.04	0.65	1.67
*p53*	−0.22	0.8	0.5	1.27

### 3.6 Survival analysis results

**FIGURE 5:**
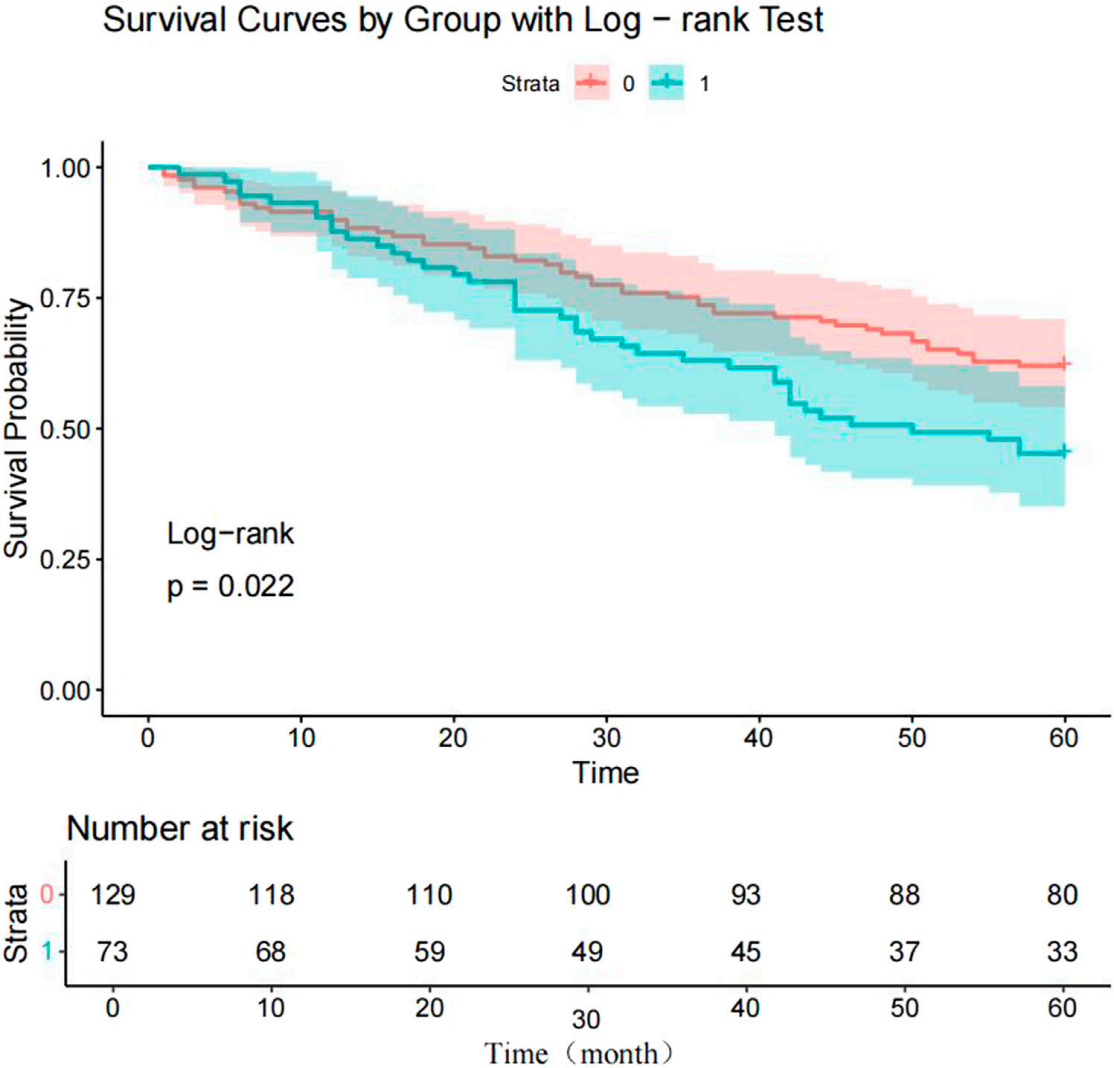
Kaplan-Meier survival curves for endometrial carcinoma (EC) patients. Note: 0 = good prognosis group; 1 = poor prognosis group.

**FIGURE 6:**
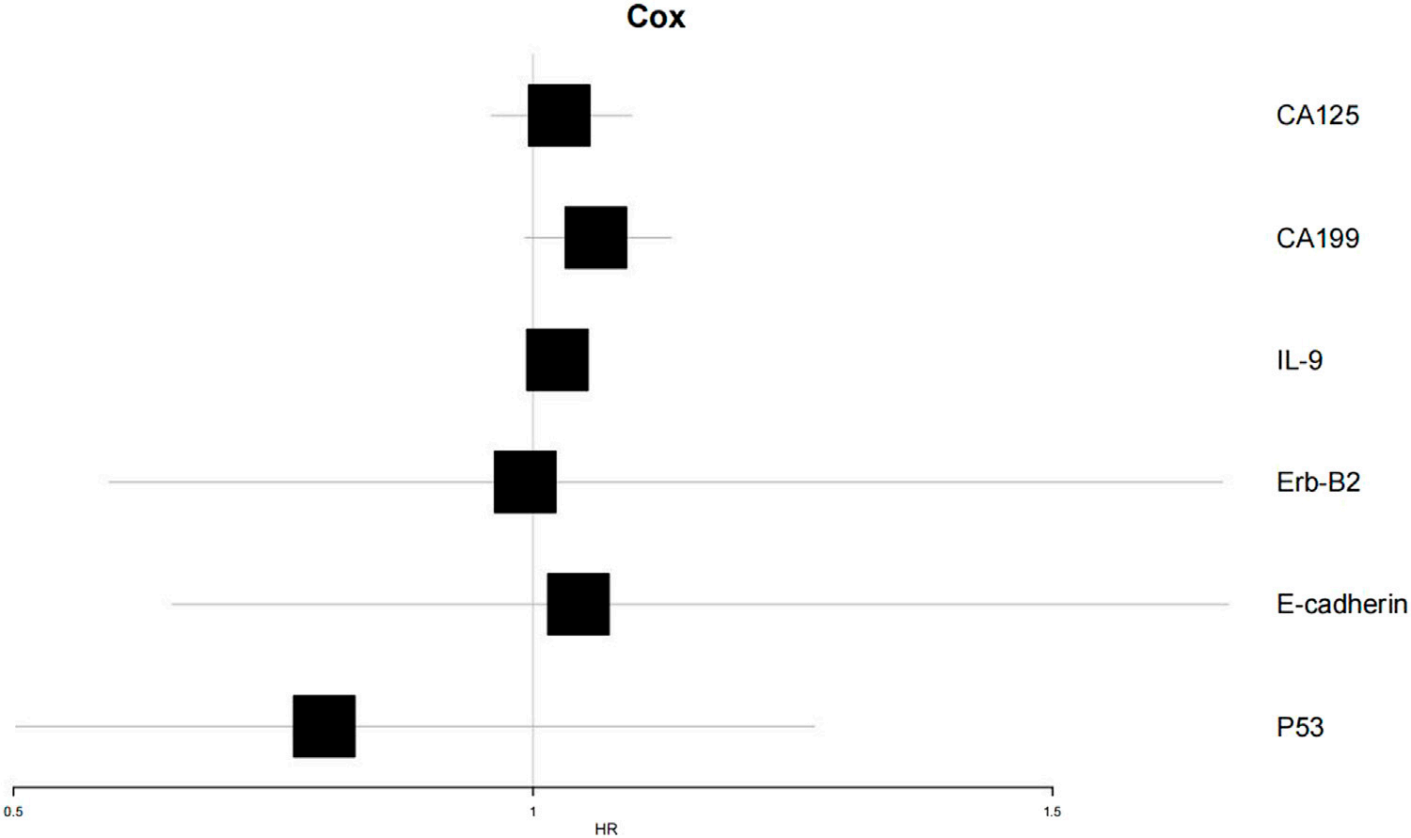
Forest plot of Cox proportional hazards regression analysis.

To verify the clinical effectiveness of prognostic grouping, K-M survival analysis was conducted. The results showed that the 5-year survival rate of the good prognostic group (62.0%–100%) was significantly higher than that of the poor prognostic group (45.2%–100%). The Log rank test confirmed that there was a statistically significant difference in survival curves between the two groups, which proves that the initial prognostic grouping has clinical significance. (*P* < 0.05; Figure 5). Cox proportional hazards regression analysis incorporating factors identified by logistic regression revealed hazard ratios (HR) < 1 for *Erb-B2* and *p53*, indicating protective effects, while *E-cadherin* exhibited an HR > 1, signifying increased risk. Clinical markers CA125, CA199, and IL-9 also showed HR > 1, correlating with elevated risk of adverse outcomes (Table 6; Figure 6).

**TABLE 6:** Subgroup analysis results.

Group		POLE mutation	Microsatellite instability	Copy number low	Copy number high	F	P
TIME		47.20 ± 21.15	44.18 ± 19.92	45.40 ± 19.79	45.88 ± 19.68	0.093	0.964
CA125		51.42 ± 3.37	51.52 ± 3.25	50.39 ± 3.60	50.94 ± 3.26	1.006	0.391
CA199		56.58 ± 3.19	55.76 ± 3.83	56.45 ± 3.51	56.33 ± 3.41	0.365	0.778
Hb		131.41 ± 11.84	132.93 ± 10.72	134.36 ± 9.16	131.14 ± 10.05	1.342	0.262
RBC		4.74 ± 0.28	4.61 ± 0.42	4.70 ± 0.39	4.65 ± 0.37	0.61	0.609
PLT		208.73 ± 10.56	205.96 ± 8.03	204.26 ± 8.77	206.73 ± 8.04	1.474	0.223
WBC		4.67 ± 0.44	4.59 ± 0.39	4.54 ± 0.35	4.65 ± 0.34	1.168	0.323
IL-9		96.27 ± 11.19	93.46 ± 10.81	93.92 ± 10.27	94.76 ± 10.30	0.288	0.834
ERBB2	Yes	5 (50%)	16 (41%)	12 (19%)	36 (40%)		0.0151^a^
ERBB2	No	5 (50%)	23 (59%)	51 (81%)	54 (60%)		
PIK3CA	Yes	5 (50%)	12 (30.8%)	30 (47.6%)	43 (47.8%)		0.2811*
PIK3CA	No	5 (50%)	27 (69.2%)	33 (52.4%)	47 (52.2%)		
CCND1	Yes	3 (30%)	22 (56.4%)	23 (36.5%)	45 (50%)		0.1351^a^
CCND1	No	7 (70%)	17 (43.6%)	40 (63.5%)	45 (50%)		
VEGF	Yes	3 (30%)	18 (46.2%)	11 (17.5%)	33 (36.7%)		0.0103^a^
VEGF	No	7 (70%)	21 (53.8%)	52 (82.5%)	57 (63.3%)		
KIT	Yes	5 (50%)	22 (56.4%)	19 (30.2%)	38 (42.2%)		0.0616*
KIT	No	5 (50%)	17 (43.6%)	44 (69.8%)	52 (57.8%)		
PTEN	Yes	5 (50%)	22 (56.4%)	42 (66.7%)	52 (57.8%)		0.573^a^
PTEN	No	5 (50%)	17 (43.6%)	21 (33.3%)	38 (42.2%)		
E-cadherin	Yes	2 (20%)	23 (59%)	36 (57.1%)	52 (57.8%)		0.1481^a^
E-cadherin	No	8 (80%)	16 (41%)	27 (42.9%)	38 (42.2%)		
P53	Yes	3 (30%)	18 (46.2%)	34 (54%)	40 (44.4%)		0.4754^a^
P53	No	7 (70%)	21 (53.8%)	29 (46%)	50 (55.6%)		
Prognostic outcomes	Good	6 (60%)	25 (64.1%)	46 (73%)	52 (57.8%)		0.2721^a^
Prognostic outcomes	Poor	4 (40%)	14 (35.9%)	17 (27%)	38 (42.2%)		

^a^ Indicates row Fisher exact test.

### 3.7 Construction of random forest model

**FIGURE 7:**
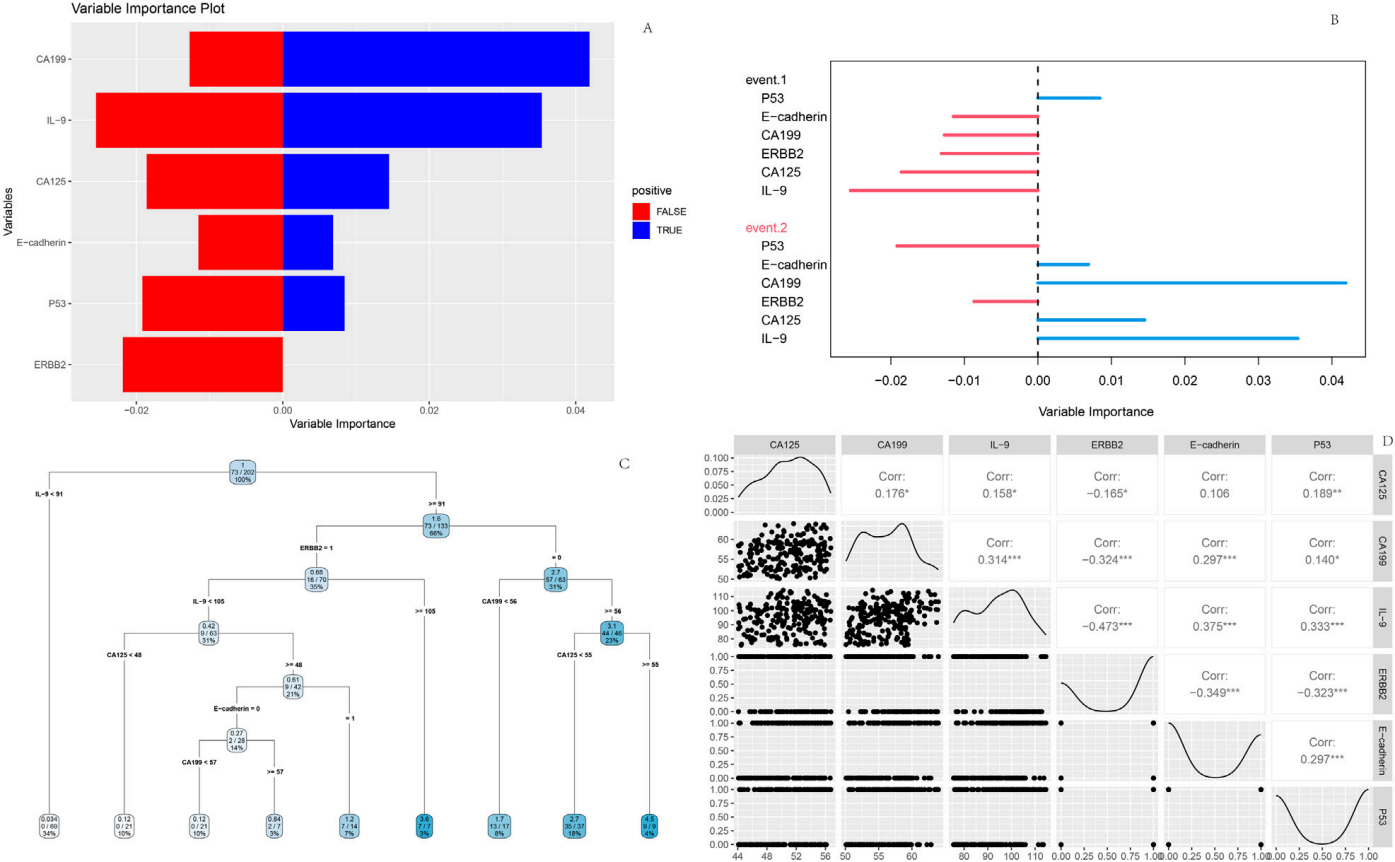
Random Forest model outputs **(A)** Feature importance plot; **(B)** Variable importance plot; **(C)** Decision tree; **(D)** Variable interaction matrix.

A random forest model was constructed using the screened clinical indicators and genetic markers. Feature importance analysis revealed that CA199 exhibited relatively prominent positive importance in the model, suggesting its potential role as a key variable in correlation analyses or predictive modeling. Random forest feature importance ranking: CA199 (0.035)>IL-9 (0.028)>CA125 (0.022)>Erb-B2 (0.018); The decision tree is based on clinically detectable indicators to construct a hierarchical rule (Figure 7C) when CA199 > 56.8 U/mL (the clinical routine detection threshold is about 37 U/mL), the risk of poor prognosis is 2.3 times higher than below the threshold (68.7% vs. 29.4%); If both CA199 > 56.8 U/mL and CA125 > 51.2 U/mL are met, the risk of poor prognosis further increases to 72.3% (much higher than the overall poor prognosis rate of 36.1%) In contrast, *E-cadherin*, *p53*, and *Erb-B2* displayed balanced but lower importance scores, indicating weaker contributions to outcome prediction. Directional importance analysis demonstrated variability in the magnitude and direction of variable impacts across events. CA199 showed higher positive importance for Event two (poor prognosis, represented by longer blue bars), whereas IL-9 exerted notable negative importance for Event 1 (favorable prognosis, indicated by longer red bars).

The decision tree diagram illustrated branching rules based on thresholds of CA125 and CA199 levels. Starting from the root node, data partitioning proceeded through sequential splits determined by these biomarkers, ultimately forming terminal leaf nodes for outcome classification. Interaction matrix analysis highlighted strong negative correlation (r = −0.473) between *Erb-B2* and IL-9, reflecting their interconnected roles in prognostic stratification (Figure 7).

## 4 Discussion

EC, one of the most common malignancies in the female reproductive system, poses a significant threat to patient health. Beyond causing debilitating symptoms such as abnormal vaginal bleeding, discharge, and pain that severely impair quality of life, EC progression often involves local invasion and distant metastasis, markedly increasing complications and mortality risks (Gao et al., 2025). Current surgical interventions for EC, including comprehensive staging surgery, extrafascial hysterectomy, and laparoscopic procedures, yield variable prognoses influenced by tumor stage, histopathological subtype, and therapeutic approach (Cao et al., 2025). Given this severe disease burden and complex clinical management and prognostic landscape, our study employed bioinformatics to identify DEGs in EC and further constructed Cox proportional hazards and random forest models, providing clinicians with comprehensive and precise tools to better understand patient conditions and improve prediction of poor prognosis.

Through bioinformatics analysis, this study first screened significant DEGs, then selected feature genes via LASSO regression, and investigated their associations with the prognosis of EC patients. The results suggest a potential unique regulatory mechanism for *Erb-B2* in EC, hypothesizing that *Erb-B2* may delay disease progression and improve prognosis by activating certain tumor-suppressive signaling pathways or inhibiting the expression of proteins involved in tumor cell invasion and metastasis (Ye et al., 1996). Subsequent research could validate its specific mechanisms through cellular function experiments and explore whether enhancing *Erb-B2* expression or activity could lead to the development of novel therapeutic strategies. In contrast, *E-cadherin3* and *p53*, identified as risk genes, exhibited abnormal expression that negatively impacted EC prognosis. As a member of the cell adhesion molecule family, downregulated *E-cadherin3* may disrupt intercellular junctions, enabling tumor cells to breach the basement membrane and undergo invasion and metastasis (Adzraku et al., 2023). Additionally, *p53* showed an odds ratio (OR) of 12.372, indicating a strong association with EC prognosis, consistent with Bourdon’s findings (Bourdon, 2007). *p53* is a critical tumor-suppressor gene in humans. Under normal physiological conditions, p53 protein responds to intracellular stress signals such as DNA damage (Wang et al., 2023), regulating the expression of downstream target genes to induce cell cycle arrest, DNA repair, or apoptosis, thereby maintaining genomic stability (Liu et al., 2024). However, during tumorigenesis, *p53* frequently undergoes mutation (Kennedy and Lowe, 2022), and mutant *p53* not only loses its original tumor-suppressive functions but may also acquire new pro-cancer functions (Chen et al., 2022). In EC, the high OR value of *p53* suggests widespread mutation, with mutant *p53* potentially worsening prognosis through multiple pathways. Clinically, CA125, CA199, and IL-9, identified as risk factors for poor prognosis, align with previous research on tumor markers and inflammatory cytokines in tumor progression (Zhao et al., 2021). The molecular subtyping analysis of our EC cohort, based on TCGA classification (POLE mutation, MSI, CNL, and CNH), revealed critical associations between subtype-specific characteristics, key gene expression, and clinical outcomes, enriching our understanding of EC heterogeneity. Notably, the CNL subtype exhibited the highest proportion of favorable prognosis (73%), consistent with prior observations that CNL is associated with better clinical outcomes due to its lower genomic instability and reduced aggressive features (Onoprienko et al., 2024). These indicators not only reflect tumor burden but may also participate in regulating the tumor microenvironment (Kartikasari et al., 2021). Therefore, dynamic monitoring of their levels can help clinicians timely assess treatment efficacy and adjust interventions.

Survival and random forest models constructed using the selected genes and clinical indicators demonstrated promising clinical utility. Survival curves showed significant differences in survival outcomes between EC patients with good and poor prognoses, providing an intuitive basis for initial clinical prognostic assessment. If there is a lack of K-M analysis to validate the effectiveness of grouping, the subsequent association analysis of the model for ‘prognostic grouping’ will lose its clinical basis. Therefore, K-M analysis is a key validation step that connects clinical grouping with statistical models. Clinically, CA19-9 could serve as a core indicator for prognostic evaluation in EC patients. Regular monitoring of its levels, combined with CA125 and IL-9, would enable dynamic assessment of disease progression and treatment response. Patients with abnormally elevated marker levels require close vigilance for poor prognosis risks and prompt treatment adjustments. Meanwhile, genetic testing for *Erb-B2*, *p53*, and *E-cadherin* in newly diagnosed EC patients can clarify their genetic status. For those with *Erb-B2* overexpression or *p53* mutation, combined targeted therapies (e.g., anti-*Erb-B2* monoclonal antibodies) could be considered; for patients with *E-cadherin* expression loss, strategies to restore its function, such as immunomodulatory therapy, warrant exploration.

From a clinical practice perspective, the results of the random forest model have clear translational value: (1) CA199, CA125, and IL-9 are all routine serum testing indicators in clinical practice, which can be carried out in primary hospitals For preoperative patients, these three indicators can be used to quickly screen high-risk populations, and more intensive postoperative follow-up is recommended as a priority. (2) For patients with high levels of CA199 and CA125, preoperative neoadjuvant therapy (such as chemotherapy combined with anti angiogenic drugs) can be considered to reduce tumor burden and postoperative recurrence; (3) Although the importance of Erb-B2 is relatively low, its inclusion in the model as a target for approved targeted drugs (such as trastuzumab) provides a basis for precise stratification and targeted therapy. For example, for patients with Erb-B2 positive and CA199 normal, postoperative combined anti-Erb-B2 treatment can further reduce the risk of recurrence and avoid overtreatment (Wang et al., 2022).

## 5 Conclusion

In conclusion, this study successfully identified key DEGs through bioinformatics analysis and constructed a Cox proportional hazards model and a random forest model, providing important genetic targets and theoretical evidence for the early diagnosis and prognostic assessment of EC. These findings empower clinicians to predict prognosis more accurately and develop personalized treatment plans. However, this single-center study may produce selection bias, necessitating further validation through *in vitro* and *in vivo* experiments targeting these key genes (*Erb-B2*, *p53* and *E-cadherin*) to explore innovative therapeutic strategies and improve treatment outcomes and quality of life for EC patients.

## Data Availability

The original contributions presented in the study are included in the article/supplementary material, further inquiries can be directed to the corresponding author.
